# The effectiveness of genomic selection for milk production traits of Holstein dairy cattle

**DOI:** 10.5713/ajas.19.0546

**Published:** 2019-12-24

**Authors:** Yun-Mi Lee, Chang-Gwon Dang, Mohammad Z. Alam, You-Sam Kim, Kwang-Hyeon Cho, Kyung-Do Park, Jong-Joo Kim

**Affiliations:** 1 Department of Biotechnology, Yeungnam University, Gyeongsan 38541, Korea; 2 Division of Animal Breeding and Genetics, National Institute of Animal Science, RDA, Cheonan 31000, Korea; 3 Korea National College of Agriculture and Fisheries, Jeonju, 54874, Korea; 4 Department of Animal Biotechnology, Chonbuk National University, Jeonju 54896, Korea

**Keywords:** Generation Interval, Genomic Estimated Breeding Value (GEBV), Heritability, Milk Production Traits, Reliability

## Abstract

**Objective:**

This study was conducted to test the efficiency of genomic selection for milk production traits in a Korean Holstein cattle population.

**Methods:**

A total of 506,481 milk production records from 293,855 animals (2,090 heads with single nucleotide polymorphism information) were used to estimate breeding value by single step best linear unbiased prediction.

**Results:**

The heritability estimates for milk, fat, and protein yields in the first parity were 0.28, 0.26, and 0.23, respectively. As the parity increased, the heritability decreased for all milk production traits. The estimated generation intervals of sire for the production of bulls (L_SB_) and that for the production of cows (L_SC_) were 7.9 and 8.1 years, respectively, and the estimated generation intervals of dams for the production of bulls (L_DB_) and cows (L_DC_) were 4.9 and 4.2 years, respectively. In the overall data set, the reliability of genomic estimated breeding value (GEBV) increased by 9% on average over that of estimated breeding value (EBV), and increased by 7% in cows with test records, about 4% in bulls with progeny records, and 13% in heifers without test records. The difference in the reliability between GEBV and EBV was especially significant for the data from young bulls, i.e. 17% on average for milk (39% vs 22%), fat (39% vs 22%), and protein (37% vs 22%) yields, respectively. When selected for the milk yield using GEBV, the genetic gain increased about 7.1% over the gain with the EBV in the cows with test records, and by 2.9% in bulls with progeny records, while the genetic gain increased by about 24.2% in heifers without test records and by 35% in young bulls without progeny records.

**Conclusion:**

More genetic gains can be expected through the use of GEBV than EBV, and genomic selection was more effective in the selection of young bulls and heifers without test records.

## INTRODUCTION

With the development of DNA analysis technology and the reduced cost of single nucleotide polymorphism (SNP) chip analysis, a lot of research has been conducted on the genomic selection of dairy cattle [1–7]. Gengler et al [8] proposed an algorithm that could predict genomic information about individuals without genomic information, and VanRaden [9] developed methods to calculate the genomic relationship matrix and to estimate the genomic estimated breeding value (GEBV). Misztal et al [10] later proposed a new algorithm by combining existing pedigree information and genomic information. Recently, Liu et al [11] developed the SNP single-step genomic model and presented methods for estimating effects of SNPs directly from the analysis model. The use of the GEBV of dairy cattle was formalized from August, 2010 in Germany, and young bulls without daughters can be selected as ‘proven’ bulls, while those that have been selected by GEBV are called ‘genomic’ bulls [12].

In the USA and Canada, young bulls have been evaluated using genomic information since 2009. For individuals evaluated in these countries without phenotypic information, it has been reported that the GEBV estimated using genomic information was more reliable than the estimated breeding value (EBV) estimated from the conventional best linear unbiased prediction (CBLUP) method [13]. In Japan, a reference population of about 4,000 young bulls was established and genomic information has since been applied in the juvenile selection of young bulls and heifers in the Japanese population [14].

Schaeffer [15] proposed the multiple-trait across country evaluation (MACE) project, in which 35 countries, including the Republic of Korea, are now participating [12]. Sullivan and VanRaden [16] proposed the genomic MACE (GMACE) project, which uses genomic information in the evaluation of cattle and has been in operation since 2014. Korea is currently establishing the reference populations and accumulating the genomic data it needs to participate in GMACE. The purpose of the present study was to test the efficiency of genomic selection for milk production traits in the domestic population of dairy cattle in Korea.

## MATERIALS AND METHODS

### Single nucleotide polymorphism data

A total of 2,090 head of cattle, consisting of both bulls (507 head) and cows (1,583 head), were genotyped using a Bovine SNP50k chip (Illumina, San Diego, CA, USA), through which 50,908 SNPs were identified. To ensure the quality of the genotypic data obtained, SNPs were excluded from analyses if they were found on the sex chromosomes, lacked chromosomal information, had missing rates with higher than 10%, lacked polymorphism (all homo- or heterozygous), had a minor allele frequency less than 1%, or were found with a chi-squared value of the Hard-Weinberg disequilibrium greater than 23.9 (p<1.0×10^−6^). Animals with SNP missing rates greater than 10% were also excluded from analyses. After the quality control tests, 2,007 individuals and 41,837 SNPs were used in the following analysis (Supplementary Table S1).

### Milk production data

Based on the test records for the dairy cows calved from 2002 to 2016, individuals were excluded from analyses if their records exceeded the following bounds: 305-days milk yield outside the range of 2,500 to 16,000 kg, 305-days fat yield outside the range of 70 to 600 kg, 305-days protein yield outside the range of 80 to 500 kg, for cows exceeding third parity. Additionally, data from cows were not used for analyses for whom less than 5 records were recorded within one herd-year-season (HYS), or whose calving ages were outside the range of 17 to 31 months in the first parity, 31 to 45 months in the second parity, or 45 to 59 months in the third parity. These eliminations were due to the potential outliers or ambiguous parity. Therefore, a total of 506,481 milk production records from 293,855 animals were used for the final analyses (Supplementary Table S2).

### Statistical model

The HYS and parity×month of age at calving (PA) were included as fixed effects in a statistical analysis that used the following model:

$$y_{i}=X_{i}b_{i}+Z_{i}a_{i}+e_{i}$$

Where *y_i_* = n×1 vector of observation in the ith parity, *b_i_* = *p*×1 vector of the fixed effect, *a_i_* = *q*×1 vector of the additive random genetic effect, *e_i_* = n×1 vector of the residual effect, and *X_i_*(n×p), *Z_i_*(n×q), and *W_i_*(n×q) were known incidence matrices corresponding to *b_i_*, and *a_i_*, respectively. The total numbers of HYS, PA, and animals within pedigree values included in the analysis using this model, were 62,287, 75, and 384,406 head, respectively. Since there were no observed values comparable each parity by trait value, the value of the covariance matrix was set equal to zero in the matrix of the error variance and covariance shown below:

$$\begin{array}{ll} R\; & =\left[\begin{array}{ccc} \sigma_{e1}^{2} & 0 & 0\\ 0 & \sigma_{e2}^{2} & 0\\ 0 & 0 & \sigma_{e3}^{2} \end{array}\right]\\ & =diag(r_{11},r_{22},r_{33}),R^{-1}=diag(r^{11},r^{22},r^{33}) \end{array}$$

The GEBV was estimated using Single-Step genomic best linear unbiased prediction which integrates the genomically derived relationships with pedigree relationships [10].

The mixed model equation (MME) used in further analyses was as follows:

$$\left[\begin{array}{cccc} r^{11}{X_{1}}^{\prime }X_{1} & 0 & r^{11}{X_{1}}^{\prime }Z_{1} & 0\\ 0 & r^{22}{X_{2}}^{\prime }X_{2} & 0 & r^{22}{X_{2}}^{\prime }Z_{2}\\ r^{11}{Z_{1}}^{\prime }X_{1} & 0 & r^{11}{Z_{1}}^{\prime }Z_{1}+H^{-1}g^{11} & H^{-1}g^{12}\\ 0 & r^{22}{Z_{2}}^{\prime }X_{2} & H^{-1}g^{21} & r^{22}{Z_{2}}^{\prime }Z_{2}+H^{-1}g^{22} \end{array}\right]\;\left[\begin{array}{c} \hat{b_{1}}\\ \hat{b_{2}}\\ \hat{a_{1}}\\ \hat{a_{2}} \end{array}\right]=\left[\begin{array}{c} r^{11}{X_{1}}^{\prime }y_{1}\\ r^{22}{X_{2}}^{\prime }y_{2}\\ r^{11}{Z_{1}}^{\prime }y_{1}\\ r^{22}{Z_{2}}^{\prime }y_{2} \end{array}\right]$$

where *g*_11_, *g*_22_ = genetic variance, *g*_12_, *g*_21_ = genetic covariance, *r*_11_, *r*_22_ = error variance, *r*_12_, *g*_21_ = error covariance, $H^{-1}=A^{-1}+\left[\begin{array}{cc} 0 & 0\\ 0 & G^{-1}-A_{22}^{-1} \end{array}\right]$, *A*^−1^ = the inverse matrix of the numerator relationship matrix, *G*^1−^ = the inverse matrix of the genomic relationship matrix, and $A_{22}^{-1}$ = the inverse matrix of the numerator relationship matrix of dairy cattle with genomic information. The reliability (*r*^2^) of breeding value was calculated using the prediction error variance (PEV) value by the following formula: $r^{2}=1-(PEV/\sigma_{a}^{2})$.

$$\begin{array}{l} Integrated\;BV_{i}=1^{st}BV_{i}\times 0.5+2^{nd}BV_{i}\times 0.3+3^{rd}BV_{i}\times 0.2\\ \left(BV_{i}=EBV\;or\;GEBV\;of\;the\;i^{th}\;trait\right) \end{array}$$

Variance components, and EBV and GEBV values were es timated using the BLUPF90 family program [17].

## RESULTS

### Generation interval

In the domestic population of dairy cattle examined, the estimated generation intervals of sire for the production of bulls (L_SB_) and that for the production of cows (L_SC_) were 7.9 and 8.1 years, respectively, and the estimated generation intervals of dams for the production of bulls (L_DB_) and cows (L_DC_) were 4.9 and 4.2 years, respectively (Table 1).

### Genetic parameters

The estimated heritability of milk yield by parity in the first, second, and third parity were 0.28, 0.20, and 0.16, respectively, while that for fat yield were 0.26, 0.23, and 0.20, and that for protein yield were 0.23, 0.18, and 0.15, respectively (Table 2).

### Estimated breeding value and genomic estimated breeding value

The overall regression coefficient estimates between EBV and GEBV for all milk production data analyzed were 0.9075 for milk, 0.9202 for fat, and 0.9012 for protein yields. The regression coefficient estimates between EBV for GEBV for the cows with test records and bulls with progeny records were in the ranges of 0.9210 to 0.9511 and 0.9378 to 0.9519, respectively, while those for bulls without progeny records were the lowest and in the range of 0.5348 to 0.6047 (Supplementary Table S3).

### Reliability

When genomic information was used, the reliability of trait selection increased by 9% on average in the overall data set when compared to the method using only pedigree information; the reliability was similarly increased by using genetic information by 7% for cows with test records, 4% for bulls with progeny records, 13% for heifers without test records, and 17% for young bulls without progeny records (Table 3).

### Genetic gain

When selected using genetic information, the genetic gains in milk yield for the cows with test records increased by about 7.1%, over the gains achieved with CBLUP methods, and gains similarly increased by about 2.9% for bulls with progeny records, 24.2% for heifers without test records, and 35% for bulls without progeny records (Table 4). Compared with the CBLUP method, the genetic gains in fat yield were increased by about 7.7% in cows with test records and 2.7% in bulls with progeny records, while gains increased by about 23.6% for heifers without test records and 33.3% for bulls without progeny records (Supplementary Table S4). The genetic gains in protein yield increased by about 8.57% in cows with test records and 2.8% in bulls with progeny records over gains with the CBLUP method, while gains were increased by about 23.4% for heifers without test records and 34.6% for bulls without progeny records (Supplementary Table S5).

## DISCUSSION

The heritability estimates for milk, fat, and protein yields in the first parity in this study were 0.28, 0.26, and 0.23, respectively; these results have been reported to Interbull. As the parity increased, the heritability decreased for all milk production traits. The genetic correlation coefficients among parities for milk, fat and protein yields were in the range of 0.85 to 0.99, while the phenotypic correlation coefficients among parities were lower than the genetic correlation coefficients and in the range of 0.42 to 0.52. Similar results to these were previously reported in other countries [18,19].

In the Korean dairy cattle population examined, the esti mated L_SB_, L_SC_, L_DB_, and L_DC_ were 7.9, 8.1, 4.9, and 4.2 years, respectively. For the Holstein population in the USA, the generation intervals for the L_SB_, and L_SC_ reported in 2010 (before genomic selection was applied) were about 7 years, and those for the L_DB_ and L_DC_ were about 4 years. After genomic selection had been applied for 5 years, the generation intervals for the L_SB_, L_SC_, L_DB_, and L_DC_ were reported to decrease to about 3, 5, 3, and 3.6 years, respectively [5]. In Canada, the average generation interval for Holstein cattle was 7 years in the 1970s, and since then it has decreased to about 5.8 years. For L_DC_ it was 4.2 years and remained stable around this value [20].

When the regression coefficients between GEBV estimated from the single-step best linear unbiased prediction (ssBLUP) method and EBV estimated from the CBLUP method were compared, the coefficients estimated for young bulls without progeny records were the lowest, and in the range of 0.54 to 0.61. It can thus be concluded that genomic selection was more efficient in heifers and in young bulls without test records [21].

The reliability of GEBV was higher than that of EBV, es pecially for animals without phenotypic data. These results agreed with those of Forni et al [22], who reported that the accuracy of selection was increased by using genomic information compared with that using only pedigree information. In this study it was found that selection was relatively more accurate in young bulls and heifers without phenotypic data, and the accuracy of selection increased even more when genomic information was used.

The reason for the increased accuracy resulting from using genomic information might be due to the fact that when doing this the pedigree coefficient matrix used in the CBLUP method was replaced by a genomic relationship matrix, which was derived from the genotype similarity calculated for all markers and considering Mendelian sampling [23,24]. In the overall data set, the reliability of GEBV increased by 9% on average over that of EBV, and increased by 7% in cows with test records, about 4% in bulls with progeny records, and 13% in heifers without test records. The difference in the reliability between GEBV and EBV was especially great for data from young bulls, as this increased by 17% on average for milk (39% vs 22%), fat (39% vs 22%), and protein (37% vs 22%) yields. Similar results were obtained by VanRaden et al [13] who reported that in the USA’s Holstein population combined genomic predictions had realized reliabilities that were 23% greater than reliabilities of parent averages (50% vs 27%) when averaged across all traits. These results suggested that genomic selection was more effective in the selection of young bulls and heifers without test records [21].

In other studies that compared the reliability of genomic and conventional selection methods for the estimation of breeding values, the reliability of GEBV was comparable to that of either the parent average or the pedigree index method [25–27]. These types of comparisons are possible since the reliability of genomic selection is very high for the selection of young bulls without test records for their daughters. Compared with conventional selection methods, genomic selection can accelerate the improvement of animals, since the reliability of genomic selection is relatively high and it can be used to reduce generation intervals. Therefore, genomic selection can be efficiently used for the juvenile selection of dairy cattle.

For the selection of proven bulls in Korea, first about 40 head of young bulls are selected and then 2 of them are further selected on the basis of the progeny test records from 20 of their daughter heifers. For the selection of young heifers, pedigree information is used. Therefore, the selection rate of young bulls is 5% and the selection intensity (i) is 2.06, while for young heifers the selection rate is 90% (9 out of 10) and the selection intensity (i) is 0.20 [28].

When selected for the milk yield using GEBV, the genetic gain increased in this study by about 7.1% over the gain with the EBV method in cows with test records, and by 2.9% in bulls with progeny records, while it increased by about 24.2% in heifers without test records and by 35% in young bulls without progeny records. Therefore, the application of genomic selection to gene introgression can help to speed up the process of introgression of a gene while simultaneously increasing the genetic gain [3].

Since the selection intensity actively used in the domestic population in Korea was applied in the present study, more genetic gains to this population can be expected through the use of genomic selection, since more young bulls and heifers can be selected to improve desirable traits.

Wiggans et al [26] reported that during the genomic selec tion of cattle conducted in 2011 in the USA, the reliability of the selection of milk yield increased by 34.0% over the parent average, and that of fat and protein yields increased by 33.8% and 24.9%, respectively, indicating that reliabilities can be increased even more than those we obtained in our study. The smaller improvements we found might have been due to the relatively very small reference population we used [29,30]. When genomic selection is applied in the selection of dairy cattle in the domestic population, the size of the reference population will increase continuously and potentially result in greater improvements, but this will take time.

Therefore, through the participation of Korea in interna tional genetic performance evaluation programs using genomic information, or by sharing data with overseas dairy cattle populations related to the genetic resources of domestic dairy cattle populations, the improvement of dairy cattle can be facilitated. Also, the efficiency of data utilization should be increased and the introduction of new technologies should be accelerated in Korea to facilitate dairy cattle improvement.

## Supplementary Data



## Figures and Tables

**Figure 1:**
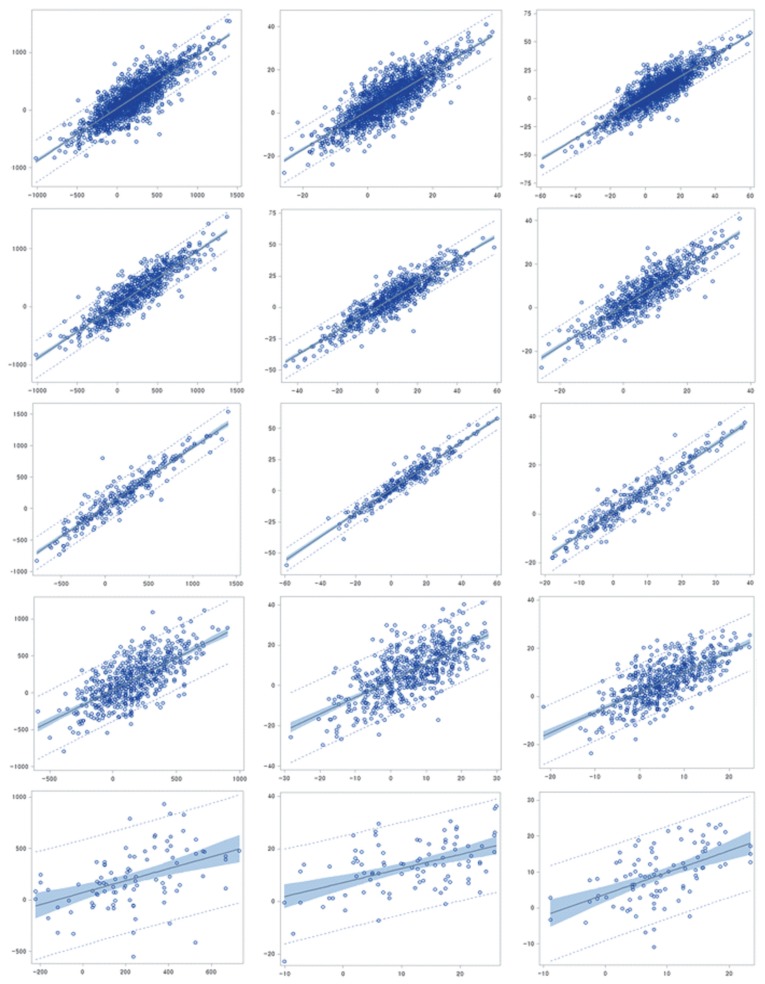
Relationship between breeding value (EBV) and genomic breeding value (GEBV) for milk, fat protein yields (left; milk, center; fat, right; protein) in each group (from top to bottom; overall, cows with record, sires with progeny, heifers without record and bulls without progeny).

**Table 1:** Generation intervals in Holstein dairy cattle population

Pathway	No. of pairs	Generation interval (yr)			

		Mean±STD	Skewness	Median	Mode
Sire → offspring (L_SO_)	810,391	8.10±1.99	1.21	7.85	6.92
Sire → Bull (L_SB_)	5,694	7.93±2.59	1.12	7.46	6.32
Sire → Cow (L_SC_)	804,697	8.10±1.98	1.12	7.86	6.92
Dam → offspring (L_DO_)	780,239	4.20±2.02	1.97	3.76	2.00
Dam → Bull (L_DB_)	5,340	4.94±2.31	1.52	4.56	2.25
Dam → Cow (L_DC_)	774,899	4.19±2.02	1.97	3.75	2.00
Parent →offspring (L_PO_)	1,590,630	6.19±2.80	0.55	6.55	2.00

STD, standard deviation; Lso, generation interval from sire to offspring, etc.

**Table 2:** Heritabilities, standard errors, genetic and phenotypic correlations among between parities for milk production traits in a Korean Holstein cattle population

Parity	Milk production traits (kg)								

	Milk			Fat			Protein		
		
	1	2	3	1	2	3	1	2	3
1	0.28	0.51	0.43	0.26	0.52	0.44	0.23	0.51	0.42
2	0.91	0.20	0.48	0.94	0.23	0.49	0.91	0.18	0.50
3	0.88	0.99	0.16	0.90	0.99	0.20	0.85	0.98	0.15

Diagonal, heritability; upper triangle, phenotypic; lower triangle, genetic correlation.

**Table 3:** Reliabilities on GEBVs (EBVs) and standard deviations of animals with SNP information for milk production traits (kg) in each group

Groups	Traits	Parity		

		1	2	3
Overall	Milk	0.50 (0.41)±0.16	0.47 (0.39)±0.15	0.46 (0.37)±0.15
	Fat	0.50 (0.41)±0.16	0.49 (0.40)±0.15	0.47 (0.38)±0.15
	Protein	0.48 (0.39)±0.16	0.45 (0.37)±0.15	0.43 (0.34)±0.15
Cows with record	Milk	0.52 (0.45)±0.06	0.49 (0.43)±0.07	0.47 (0.41)±0.07
	Fat	0.52 (0.45)±0.06	0.51 (0.44)±0.07	0.49 (0.42)±0.07
	Protein	0.49 (0.42)±0.06	0.47 (0.40)±0.07	0.44 (0.37)±0.07
Sires with progeny	Milk	0.75 (0.71)±0.18	0.70 (0.66)±0.18	0.68 (0.64)±0.18
	Fat	0.74 (0.71)±0.19	0.72 (0.68)±0.18	0.69 (0.65)±0.18
	Protein	0.72 (0.69)±0.19	0.68 (0.64)±0.19	0.64 (0.60)±0.18
Heifer without record	Milk	0.38 (0.25)±0.06	0.36 (0.23)±0.06	0.34 (0.22)±0.06
	Fat	0.38 (0.25)±0.06	0.37 (0.24)±0.06	0.35 (0.23)±0.06
	Protein	0.36 (0.24)±0.06	0.34 (0.22)±0.06	0.32 (0.21)±0.06
Bull without progeny	Milk	0.39 (0.22)±0.07	0.37 (0.20)±0.07	0.35 (0.19)±0.07
	Fat	0.39 (0.22)±0.08	0.37 (0.21)±0.07	0.36 (0.20)±0.07
	Protein	0.37 (0.21)±0.08	0.35 (0.19)±0.07	0.33 (0.18)±0.07

GEBVs, genomic estimated breeding values; EBVs, estimated breeding values; SNP, single nucleotide polymorphisms.

**Table 4:** Genetic gains of milk yield (kg) per year by the selection method and group

Groups	Parity	*σ_a_*	*i*	*r*_*GP*_*ssBLUP*__( *r*_*GP*_*BLUP*__)	*L*	Δ*G*_1_(Δ*G*_2_)	IR
Cows with record	1	669	0.2	0.72(0.67)	4.2	23.0 (21.4)	7.5
	2	709	0.2	0.70(0.66)	4.2	23.6 (22.2)	6.7
	3	681	0.2	0.69(0.64)	4.2	22.2 (20.8)	7.1
	Mean						7.1
Sires with progeny	1	669	2.06	0.87(0.84)	8.1	147.4 (143.4)	2.8
	2	709	2.06	0.84(0.81)	8.1	150.9 (146.6)	3.0
	3	681	2.06	0.82(0.80)	8.1	142.9 (138.6)	3.1
	Mean						2.9
Heifers without record	1	669	0.2	0.62(0.50)	4.2	19.6 (15.9)	23.3
	2	709	0.2	0.60(0.48)	4.2	20.3 (16.2)	25.1
	3	681	0.2	0.58(0.47)	4.2	18.9 (15.2)	24.3
	Mean						24.2
Sires without progeny	1	669	2.06	0.62(0.47)	8.1	106.3 (79.8)	33.1
	2	709	2.06	0.61(0.45)	8.1	109.7 (80.7)	36.0
	3	681	2.06	0.59(0.44)	8.1	102.5 (75.5)	35.7
	Mean						35.0

*σ_a_*, genetic standard deviation; *i*, selection intensity; *r*_*GP*_*ssBLUP*__( *r*_*GP*_*BLUP*__), accuracy; *L*, generation interval; Δ*G*_1_ = *ssBLUP* (Δ*G*_2_ = *BLUP*), genetic gain; IR, increase rate (%).
