# Supplementary material for: The effectiveness of genomic selection for milk production traits of Holstein dairy cattle
Source: Asian-Australas J Anim Sci. 2019 Dec 24;33(3):382–9. doi: 10.5713/ajas.19.0546 (PMC7054615; doi:10.5713/ajas.19.0546)
Supplement: Supplementary file 1 [file ajas-19-0546-suppl.pdf]

Supplementary tables

Table S1

Quality control of single nucleotide polymorphism (SNP) dataset.

Description	No. of heads and SNPs
Total number of animals	2,090
Animals with missing rate over 0.10%	83
Selected animals	2,007
Total no. of SNPs	50,908
SNPs with unknown position	536
SNPs on sex chromosome (X, Y)	1,139
Total number of SNPs on autosome (1~29)	49,233
Outlier SNPs	7,396
Useful SNPs	41,837

Table S2

No. of records (percentage), cows, means and standard deviations for 305-days milk, fat and protein yields (kg) by parity

Parity	No. of records (%)	Cows (with SNP)	Heifers with SNP	Milk production traits (kg)		
				Milk	Fat	Protein
1	293,855 (58)	293,855 (789)	-	8603±1605	328±68	273±50
2	137,471 (27)	137,471 (469)	-	10048±1916	378±80	320±59
3	75,155 (15)	75,155 (279)	-	10547±1987	398±83	334±61
Overall	50,6481 (100)	293,855 (789)[1]	794	9284±1934	352±79	295±60

[1]Total number of cows, not the column sum.

Tables S3

Regression coefficients of integrated EBV to the GEBV for milk production traits in each group with SNP information.

Traits	Traits	$\beta \pm se$	r^2	t Value	Pr>|t|
Overall	Milk	0.9075±0.0137	0.72	66.05	<.0001
(1730 heads)	Fat	0.9202±0.0128	0.75	71.97	<.0001
	Protein	0.9012±0.0138	0.71	65.37	<.0001
Cows with record	Milk	0.9210±0.0166	0.80	55.35	<.0001
(749 heads)	Fat	0.9511±0.0155	0.84	61.51	<.0001
	Protein	0.9262±0.0180	0.78	51.57	<.0001
Sires with progeny	Milk	0.9400±0.0193	0.89	48.66	<.0001
(287 heads)	Fat	0.9519±0.0161	0.93	59.27	<.0001
	Protein	0.9378±0.0190	0.90	49.40	<.0001
Heifers without record	Milk	0.8638±0.0376	0.47	23.00	<.0001
(602 heads)	Fat	0.8267±0.0370	0.45	22.35	<.0001
	Protein	0.8424±0.0347	0.50	24.29	<.0001
Bulls without progeny	Milk	0.5818±0.1240	0.20	4.69	<.0001
(92 heads)	Fat	0.5348±0.0983	0.25	5.44	<.0001
	Protein	0.6047±0.1007	0.29	6.01	<.0001

Tables S4

Genetic gains of fat yield (kg) per year by the selection method and group.

group	Parity	σ_a	i	$r_{GP_{ssBLUP}}(r_{GP_{BLUP}})$	L	$\triangle G_1(\triangle G_2)$	IR
	1	25	0.2	0.72 (0.67)	4.2	0.9 (0.8)	7.5
Cows	2	30	0.2	0.71 (0.66)	4.2	1.0 (1.0)	7.7
with record	3	29	0.2	0.70 (0.64)	4.2	1.0 (0.9)	8.0
	Mean						7.7
	1	25	2.06	0.86 (0.84)	8.1	5.5 (4.4)	2.1
Sires	2	30	2.06	0.85 (0.82)	8.1	6.5 (6.3)	2.9
with progeny	3	29	2.06	0.83 (0.81)	8.1	6.2 (6.0)	3.0
	Mean						2.7
	1	25	0.2	0.62 (0.50)	4.2	0.7 (0.6)	23.3
Heifers	2	30	0.2	0.61 (0.49)	4.2	0.9 (0.7)	24.2
without record	3	29	0.2	0.59 (0.48)	4.2	0.8 (0.7)	23.4
	Mean						23.6
Bulls	1	25	2.06	0.62 (0.47)	8.1	4.0 (3.0)	33.1
without progeny	2	30	2.06	0.61 (0.46)	8.1	4.7 (3.5)	32.7
	3	29	2.06	0.60 (0.45)	8.1	4.5 (3.5)	34.2
	Mean						33.3

σ_a, genetic standard deviation; i, selection intensity; $r_{GP_{ssBLUP}}(r_{GP_{BLUP}})$, accuracy; L, generation interval; $\triangle G_1 = ssBLUP(\triangle G_2 = BLUP)$, genetic gain; IR, increase rate (%)

Tables S5

Genetic gains of protein yield (kg) per year by the selection method and group.

Group	Parity	σ_a	i	$r_{GP_{ssBLUP}}(r_{GP_{BLUP}})$	L	$\triangle G_1(\triangle G_2)$	IR
Cows with record	1	18	0.2	0.70 (0.65)	4.2	0.6 (0.5)	8.0
	2	20	0.2	0.69 (0.63)	4.2	0.7 (0.6)	8.4
	3	20	0.2	0.66 (0.61)	4.2	0.6 (0.6)	9.0
	Mean						8.5
Sires with progeny	1	18	2.06	0.85 (0.83)	8.1	3.8 (3.7)	2.2
	2	20	2.06	0.82 (0.80)	8.1	4.2 (4.1)	3.1
	3	20	2.06	0.80 (0.77)	8.1	0.4 (3.9)	3.3
	Mean						2.8
Heifers without record	1	18	0.2	0.60 (0.49)	4.2	0.5 (0.4)	22.5
	2	20	0.2	0.58 (0.47)	4.2	0.6 (0.5)	24.3
	3	20	0.2	0.57 (0.46)	4.2	0.5 (0.4)	23.4
	Mean						23.4
Bulls without progeny	1	18	2.06	0.61 (0.46)	8.1	2.7 (2.1)	32.7
	2	20	2.06	0.59 (0.44)	8.1	3.0 (2.2)	35.7
	3	20	2.06	0.57 (0.42)	8.1	2.9 (2.1)	35.4
	Mean						34.6

σ_a, genetic standard deviation; i, selection intensity; $r_{GP_{ssBLUP}}(r_{GP_{BLUP}})$, accuracy; L, generation interval; $\triangle G_1 = ssBLUP(\triangle G_2 = BLUP)$, genetic gain; IR, increase rate (%)
